# A126 ASSESSING THE QUALITY OF REFERRALS AND ADJUDICATION FOR ENDOSCOPIC RESECTION OF LARGE COLORECTAL POLYPS AT A CANADIAN TERTIARY REFERRAL CENTRE

**DOI:** 10.1093/jcag/gwac036.126

**Published:** 2023-03-07

**Authors:** Y Xiao, S Li, G May, C Teshima, J Mosko

**Affiliations:** 1 Gastroenterology and Hepatology, University of Toronto, Toronto; 2 Gastroenterology and Hepatology, University of Calgary, Calgary; 3 Gastroenterology and Hepatology, St Michaels Hospital; 4 Gastroenterology and Hepatology, University of Calgary, Toronto, Canada

## Abstract

**Background:**

Management of large colorectal polyps is increasingly complex with the expansion of endoscopic techniques, including endoscopic submucosal dissection (ESD), endoscopic mucosal resection (EMR), and endoscopic full thickness resection. Adjudicating lesions in an effort to select the optimal resection method is hugely dependent on the information included within the referral. At our institution, a referral pathway based on photo and/or video documentation was created to facilitate the timely assessment and treatment of large colorectal polyps. To date, little is known about quality of referrals for endoscopic resection of large colorectal polyps.

**Purpose:**

Our study aimed to assess the adjudication process and quality of referrals for endoscopic resection of large colorectal polyps at our advanced endoscopy referral center.

**Method:**

We conducted a single-center prospective study of consecutive colorectal polyps referred for EMR from March 2021 to March 2022. Cases selected upfront for ESD were excluded. Referral information, intraprocedural data and histology was captured. No procedural and histology data were captured if EMR does not occur after adjudication. The outcome was defined as the frequency of adequate referrals. A referral was deemed adequate if it contained: sufficient photo or video documentation, description of any characteristics that increase the difficulty of endoscopic resection, accurate polyp localization/size estimate (with <1 cm discrepancy when compared to real-time endoscopic evaluation), and description of any endoscopic features of advanced dysplasia (AD), including HGD/IMCa, or submucosal invasion (SMI).

**Result(s):**

During the study period, 213 referrals were received for colorectal polyps and underwent adjudication for EMR: 211 underwent EMR; 2 underwent ESD despite being triaged for EMR. Only 5% (10/213) of referrals were deemed to be adequate. Only 34% (73/213) contained any photo or video documentation and only 13% (28/213) photos/videos were of sufficient quality for adjudication. Difficult location or polyp characteristics, if present, were accurately described in 86.7% of referrals (183/211) of referrals. The accurate polyp location was described 80.6% of the time (170/211). Polyp size was estimated in 50.2% (107/213) of referrals. Amongst referrals with size estimated, the size was accurate in 73.8% of the time (79/107). On histological evaluation, 35.1% (74/211) of polyps had AD or SMI. Amongst polyps with AD or SMI, 48.6% (36/74) had endoscopic appearance suggestive of HGD/IMCa/SMI but only 69.4% (25/36) of these polyps with high-risk endoscopic features were accurately predicted based on the referral information.

**Conclusion(s):**

Referrals for large colorectal polyps often lack important clinical information. This significantly impairs the ability to adjudicate polyps for triage and resection and may negatively impact patient outcomes. To improve referral adequacy and patient outcomes, we plan to evaluate the impact of polyp adjudication on EMR success.

**Please acknowledge all funding agencies by checking the applicable boxes below:**

None

**Disclosure of Interest:**

None Declared

